# Preoperative risk factors associated with new focal neurological deficit and other major adverse events in first-time intracranial meningioma neurosurgery

**DOI:** 10.1007/s00701-021-04897-x

**Published:** 2021-07-14

**Authors:** Freya Sophie Jenkins, Flavio Vasella, Luis Padevit, Valentino Mutschler, Kevin Akeret, Julia Velz, Luca Regli, Johannes Sarnthein, Marian Christoph Neidert

**Affiliations:** 1 Department of Neurosurgery, University Hospital Zurich, University of Zurich, Frauenklinikstrasse 10, 8091 Zurich, Switzerland; 2 Department of Neurosurgery, Kantonsspital St. Gallen, Rorschacher Strasse 95, 9007 St. Gallen, Switzerland; 3 Clinical Neuroscience Center, University Hospital Zurich, Zurich, Switzerland

**Keywords:** Meningioma, Neurosurgery, Focal neurological deficit, Risk factors, Milan Complexity Scale, Clavien-Dindo Classification

## Abstract

**Background:**

Neurosurgical resection is the mainstay of meningioma treatment. Adverse event (AE) rates of meningioma resections are significant, but preoperative risk factors for major AEs in patients undergoing first-time meningioma surgery are largely unknown. The aim of this study was to explore major AEs and identify preoperative risk factors in patients undergoing first-time meningioma surgery.

**Methods:**

Data on all meningioma resections performed at the University Hospital Zurich from 1 January 2013 to 31 December 2018 were collected in a prospective registry. All AEs that occurred within 3 months of surgery were documented in detail and classified as “minor” or “major.” Statistical analysis included initial individual bivariate analyses of all preoperative factors and the occurrence of major AEs. Statistically significant variables were then included in a logistic regression model to identify predictors.

**Results:**

Three hundred forty-five patients were included in the study. Mean age was 58.1 years, and 77.1% of patients were female. The overall major AE rate was 20.6%; the most common of which was a new focal neurological deficit (12.8% of patients). Six preoperative factors showed a significant association with the occurrence of major AEs in bivariate analysis. All variables included in the logistic regression model showed increased odds of occurrence of major AE, but only tumor complexity as measured by the Milan Complexity Scale was a statistically significant predictor, with a score of 4 or more having twice the odds of major AEs (OR: 2.00, 95% CI: 1.15–3.48).

**Conclusion:**

High tumor complexity is an independent predictor of the occurrence of major AEs following meningioma resection. Preoperative assessment of tumor complexity using the Milan Complexity Scale is warranted and can aid communication with patients about AE rates and surgical decision-making.

## Introduction

Neurosurgical resection is the mainstay treatment for patients with intracranial meningioma [14]. Meningiomas make up roughly one-third of all primary tumors of the central nervous system (CNS) and are the most common primary intracranial tumor [21]. While most meningiomas are benign and the majority of patients can be cured by surgery alone [9, 27], the less common World Health Organization (WHO) grade II and grade III meningiomas are associated with increased mortality [4, 21]. In these patients, surgery followed by adjuvant treatment is often recommended [14]. Meningiomas of any grade can cause neurological deficits, seizures, psychological impairment, and other symptoms, leading to significant associated patient morbidity [2] and driving the need for neurosurgical treatment in many cases.

Meningioma surgery carries the risk of causing new or worsened symptomatology. Adverse events (AE) following meningioma surgery are fairly common with reported rates varying from approximately 10% to 25% [5, 10, 31, 32]. Appropriate patient selection and identification of patients at higher surgical risk are therefore of paramount importance. Consensus data on risk factors for AEs are scarce, even though many individual factors have been identified, including tumor size for new-onset seizures [31], tumor location for infection [17], surgery duration for both deep vein thrombosis and pulmonary embolism [16], as well as for mortality [7], and extent of resection according to the Simpson grade for mortality in elderly patients [3].

The aim of this study was to analyze a group of patients undergoing first-time surgery for intracranial meningioma and to identify preoperative risk factors associated with major AEs in this population in the first 3 months after surgery, using a definition of “major” that includes the onset of new focal neurological deficit.

## Methods

### Patient selection

All adult patients who underwent surgery for meningioma at the University Hospital Zurich from 1 January 2013 to 31 December 2018 were identified based on our prospective institutional registry and a complementary search of our electronic health records system [25]. The following were excluded from the total number identified: patients with spinal meningioma, patients with previous surgery at the same anatomical site as the surgery of interest, patients for whom complete records were not available, and patients who had rejected consent for research.

### Study design and recorded variables

This study is based on a combination of prospectively collected data from the neurosurgical patient registry at the University Hospital Zurich [25] with addition of retrospectively collected data from patient electronic health records.

Data for this study were collected at patient level on demographics, preoperative physical status and disability, preoperative symptomatology, preoperative radiological findings, and AEs. Tumor features such as WHO grade, histology, and brain invasion were also collected [14, 19]. Extent of resection was assessed using the Simpson grading system [26] and postoperative MRI imaging. Preoperative physical status and disability were classified according to the American Society of Anesthesiologists (ASA) classification [8] and the modified Rankin Scale (mRS) [29], respectively. Preoperative symptomatology was collected as presence versus absence of focal neurological deficit, headache, seizure, or mental alteration. Preoperative radiological findings on MRI were collected for maximum tumor diameter, tumor volume, anatomical relation to the tentorium, anatomical relation to the skull base, and scores on the Milan Complexity Scale (MCS). Scores on the MCS can be determined using preoperative imaging and cover expected major brain vessel manipulation, location in the posterior fossa, expected cranial nerve manipulation, location in an eloquent area, and tumor diameter larger than 4 cm [12]. Patients in our study were scored for each MCS component individually and the composite score was used for initial analysis.

If data from the prospective neurosurgical patient registry on AEs [25] were missing, they were supplemented with data from discharge reports, reports from the 3-month postoperative follow-up visit, and reports from visits in-between, if these had occurred. Data were initially collected for any type of AE that occurred within the 3 months after surgery, and those fulfilling the criteria as “major” were subsequently identified for inclusion in the analysis. AEs were classified as major if they were a new focal neurological deficit or if they scored grade 3a or higher on the Clavien-Dindo classification scale, thereby including AEs “requiring surgical, endoscopic or radiological intervention” (grade 3a or 3b), “life-threatening” AEs (grade 4a or 4b), and “death” (grade 5) [6]. If the same patient experienced multiple major AEs within 3 months after surgery, each major AE was counted separately.

### Statistical analysis

All variables were tabulated and analyzed by descriptive statistics. Normal distribution of quantitative data was assessed using the Shapiro–Wilk test. Analysis of association between preoperative risk factors and the occurrence of major AEs was performed in two steps. In a first step, a bivariate analysis was performed to assess the presence of a significant relationship between each preoperative variable and the occurrence of at least one major AE. The following statistical tests were used for this step of the analysis: logistic regression for normally distributed continuous quantitative variables, Pearson’s Chi-squared test for nominal dichotomous variables, and the Mann–Whitney *U* test for ordinal variables and non-normally distributed continuous quantitative variables. Yates’ continuity correction was applied to Pearson’s Chi-squared test for small samples and Fisher’s exact test was used to verify the results of small-sample comparisons.

In a second step, variables that achieved statistical significance of *p* < 0.05 in the bivariate analysis were included in a multivariate analysis using logistic regression. The variable for maximum tumor diameter was excluded from this second step, as it is also included in the MCS. The ordinal variables “score on the mRS” and “score on the MCS” were binarized for better accuracy in the logistic regression model. For the mRS, the cut-off value of 2 was used in order to distinguish absence of disability (mRS 0 or 1) from presence of disability (mRS 2 or higher) [29]. For the MCS, different cut-off values were tested in the logistic regression model in an iterative process and the cut-off value of 4 identified as most significant. Results of the multivariate analysis were reported using the odds ratio for effect size and statistical significance was defined as *p* < 0.05. Statistical analyses were performed using R Statistical Software (Version 4.0.2; R Foundation for Statistical Computing, Vienna, Austria).

## Results

### Patient characteristics and preoperative presentation

A total of 345 patients were included in this study (median age 58.6 years, interquartile range 48.3 to 67.9 years, 266 female). Of all patients, 70.7% were either healthy (ASA 1, 13.0%) or had mild systemic disease without substantive functional limitations (ASA 2, 57.7%); 66.9% of patients had no disability (mRS 0 or 1, 231 patients) with the remaining 114 patients unable to carry out at least one activity they had previously been able to perform (mRS 2 or more). Of these 114 patients, 19 had moderately severe or severe disability (mRS 4 or 5). The most common presenting symptom was focal neurological deficit, which was reported by 53.3% of patients (184 patients); 16.8% of patients in the study had experienced a seizure preoperatively (58 patients) (Table 1).

**Table 1:** Patient baseline characteristics and preoperative presentation

	Patients (*n* = 345)
Sex	
Female	266 (77.1%)
Male	79 (22.9%)
Age in years	
Mean (± SD)	58.1 (± 13.7)
Range	24–90
Physical status class	
ASA 1	45 (13.0%)
ASA 2	199 (57.7%)
ASA 3	98 (28.4%)
ASA 4	3 (0.9%)
ASA 5	0 (0.0%)
ASA 6	0 (0.0%)
Disability score	
mRS 0	67 (19.4%)
mRS 1	164 (47.5%)
mRS 2	67 (19.4%)
mRS 3	28 (8.1%)
mRS 4	10 (2.9%)
mRS 5	9 (2.6%)
mRS 6	0 (0.0%)
Symptomatology^a^	
Focal neurological deficit	184 (53.3%)
Headache	120 (34.8%)
Seizure	58 (16.8%)
Mental alteration	52 (15.1%)

^a^Percentages do not add up to 100%, as some patients reported more than one symptom

Abbreviations: *SD*, standard deviation; *ASA*, American Society of Anesthesiologists; *mRS*, modified Rankin Scale

### Radiological baseline findings and tumor location

Average maximum tumor diameter was 3.7 cm (SD ± 1.7 cm) with a range from 0.5 to 9.5 cm. Median tumor volume was 13.7 cm^3^ (interquartile range 4.5 to 34.8 cm^3^). Of patients, 76.5% had a meningioma with supratentorial location and 55.4% had a meningioma located in the skull base. Both the median as well as the most common score on the MCS was 3; 63.8% of patients had an MCS score of 3 or less and 50 patients had an MCS of 6 or more (Table 2).

**Table 2:** Radiological baseline findings

	Patients (*n* = 345)
Maximum tumor diameter in cm	
Mean (± SD)	3.7 (± 1.7)
Range	0.5–9.5
Tumor volume in cm^3^	
Median (IQR)	13.7 (4.5–34.8)
Range	0.1–180.8
Anatomical relation to tentorium	
Supratentorial	264 (76.5%)
Infratentorial	81 (23.5%)
Anatomical relation to skull base	
Skull base	191 (55.4%)
Non-skull base	154 (44.6%)
Milan Complexity Scale	
MCS 0	49 (14.2%)
MCS 1	51 (14.8%)
MCS 2	50 (14.5%)
MCS 3	70 (20.3%)
MCS 4	51 (14.8%)
MCS 5	24 (7.0%)
MCS 6	24 (7.0%)
MCS 7	17 (4.9%)
MCS 8	9 (2.6%)

Abbreviations: *SD*, standard deviation; *IQR*, interquartile range; *MCS*, Milan Complexity Scale

### Tumor features and extent of resection

Of all patients, 78.8% had a benign meningioma (WHO grade I), 19.7% had a grade II meningioma, and 1.4% had a grade III meningioma. Brain invasion was found in 7.5% of patients (26 patients). Meningothelial meningioma was the most common histological subtype and was seen in 34.5% of patients. Gross total resection identified using the Simpson grading system and defined as Simpson grades 1 to 3 [14], was achieved in 79.7% of patients (275 patients). Based on postoperative MRI imaging, 74.2% of patients showed no remaining tumor after surgery. The average extent of resection comparing preoperative and postoperative tumor volume was 92.6% across all patients.

### New focal neurological deficit and other major AEs

Of the total 345 patients, 71 (20.6%) experienced at least one major AE within the first 3 months after their meningioma surgery. Intraoperative mortality was 0%. Overall mortality within 3 months of surgery was 0.3% (one patient). This death was not attributed to the patient’s meningioma surgery.

The most common major AE was a new focal neurological deficit, which affected 12.8% of patients (Table 3). Cranial nerve palsies accounted for most new focal neurological deficits and the main affected nerves were the olfactory, optic, oculomotor, trochlear, trigeminal, abducens, and facial nerves. New-onset hemiparesis occurred in three patients (0.9%) and worsening of pre-existing hemiparesis in one patient (0.3%).

**Table 3:** Major adverse events^a^

	Patients (*n* = 345)
Major AE within 3 months of surgery	
Yes	71 (20.6%)
No	274 (79.4%)
Major AEs^b^	
New focal neurological deficit	44 (12.8%)
Intracranial hemorrhage	7 (2.0%)
Surgical site infection	5 (1.4%)
Cerebrospinal fluid leak	4 (1.2%)
Hydrocephalus	4 (1.2%)
CNS infection	3 (0.9%)
Wound dehiscence	3 (0.9%)
Cerebral infarct	2 (0.6%)
Acute heart failure	1 (0.3%)
Death	1 (0.3%)
Electrolyte disorder	1 (0.3%)
Pulmonary embolus	1 (0.3%)
Status epilepticus	1 (0.3%)
Venous sinus thrombosis	1 (0.3%)

^a^Major AEs defined as new focal neurological deficit or AE grade 3a or higher according to the Clavien-Dindo Classification [6]

^b^Sum of percentages is larger than total major AE rate as some patients experienced more than one of the major AEs listed

Abbreviations: *CNS*, central nervous system

Major AEs other than new focal neurological deficit occurred in 35 patients (9.9%). Seven patients (2.0%) experienced both a new focal neurological deficit as well as at least one additional major AE. Seven cases of major intracranial hemorrhage were reported, five of which occurred before discharge and all of which required surgical intervention under general anesthesia (Clavien-Dindo grade 3b). Of the four cases of major cerebrospinal fluid leak, one occurred before discharge and one was treated surgically without general anesthesia (Clavien-Dindo grade 3a).

Acute heart failure, major pulmonary embolism and major venous sinus thrombosis were reported in one patient each. The patient with acute heart failure was a 70-year-old male with a symptomatic skull base meningioma with a maximum diameter of 7 cm, a preoperative ASA score of 3 and a score on the MCS of 5. The patient’s acute heart failure required management on the intensive care unit (ICU) during the immediate postoperative period. The patient fully recovered and reported no lasting morbidity at the 3-month follow-up visit.

The patient with major pulmonary embolism was a 49-year-old female with a skull base meningioma, who presented preoperatively with acute worsening of neurological symptoms and whose MCS score was 7. The pulmonary embolism was non-fatal but required ICU management (Clavien-Dindo grade 4a). At 3-month follow-up, the patient had recovered fully.

In the case of major venous sinus thrombosis, the patient was a 49-year-old female with a cerebellar meningioma of the skull base. Her score on the MCS was 5. This patient also experienced a surgical site infection and wound dehiscence between discharge and 3-month follow-up, which were treated surgically under general anesthesia (Clavien-Dindo grade 3b).

### Factors associated with new focal neurological deficit and other major AEs

**Fig. 1:**
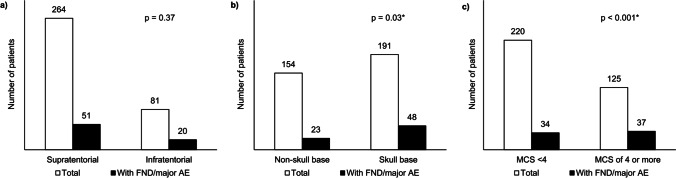
**a**–**c** Focal neurological deficits (FND) and other major adverse events (AE). **a** FNDs and other major AE by supra- vs. infratentorial location. **b** FNDs and other major AE by tumor location in skull base. **c** FNDs and other major AE by score on the Milan Complexity Scale. Abbreviations: FND, focal neurological deficit; AE, adverse event; MCS, Milan Complexity Scale. Note: *p < 0.05, statistical significance in bivariate analysis

The following six preoperative factors showed a statistically significant difference between the group with at least one new focal neurological deficit or other major AE and the group without major AEs: higher mRS score at admission, presence of focal neurological deficit at admission, presence of mental alteration at admission, larger maximum tumor diameter, tumor located in the skull base, and a higher MCS score. There was no significant difference between the two groups for sex, age, multimorbidity as measured by the ASA score, presence of headache or seizure prior to admission, tumor volume, or supratentorial versus infratentorial location (Fig. 1 “Focal neurological deficits (FND) and other major adverse events (AE)” and Table 4).

**Table 4:** Preoperative factors and occurrence of major adverse events^a^

	No major AE (*n* = 274)	At least 1 major AE (*n* = 71)	*p* value	Test statistic
Preoperative patient characteristics				
Sex (% female)	75.9%	81.7%	0.38	*χ*^2^ = 0.76
Age in years (mean)	58.3	57.2	0.55	Est. = − 0.01
Physical status (%)			0.62	*r* = 0.03
ASA 1	13.9%	9.9%		
ASA 2	56.9%	60.6%		
ASA 3	28.5%	28.2%		
ASA 4	0.7%	1.4%		
ASA 5	0.0%	0.0%		
ASA 6	0.0%	0.0%		
Disability (%)			0.01*	*r* = 0.14
mRS of 0 or 1	70.1%	54.9%		
mRS of 2 or above	29.9%	45.1%		
Symptomatology				
Focal neurological deficit (% with)	50.0%	66.2%	0.02*	*χ*^2^ = 5.31
Headache (% with)	35.0%	33.8%	0.96	*χ*^2^ = 0.00
Seizure (% with)	17.9%	12.7%	0.39	*χ*^2^ = 0.75
Mental alteration (% with)	12.8%	23.9%	0.03*	*χ*^2^ = 4.66
Radiological findings				
Maximum diameter in cm (mean)	3.7	4.2	0.02*	Est. = 0.17
Tumor volume in cm^3^ (median)	12.4	21.6	0.06	*r* = 0.10
Infratentorial location (%)	22.3%	71.8%	0.37	*χ*^2^ = 0.79
Skull base location (%)	52.2%	67.6%	0.03*	*χ*^2^ = 4.82
Milan Complexity Scale			0.00*	*r* = 0.21
Score of less than 4 (%)	67.9%	47.9%		
Score of 4 or above (%)	32.1%	52.1%		

^a^Focal neurological deficit or grade 3a or higher according to the Clavien-Dindo Classification [5]

Abbreviations: *ASA*, American Society of Anesthesiologists; *mRS*, modified Rankin Scale; *AE*, adverse events

Note: *p < 0.05. statistical significance

**Fig. 2:**
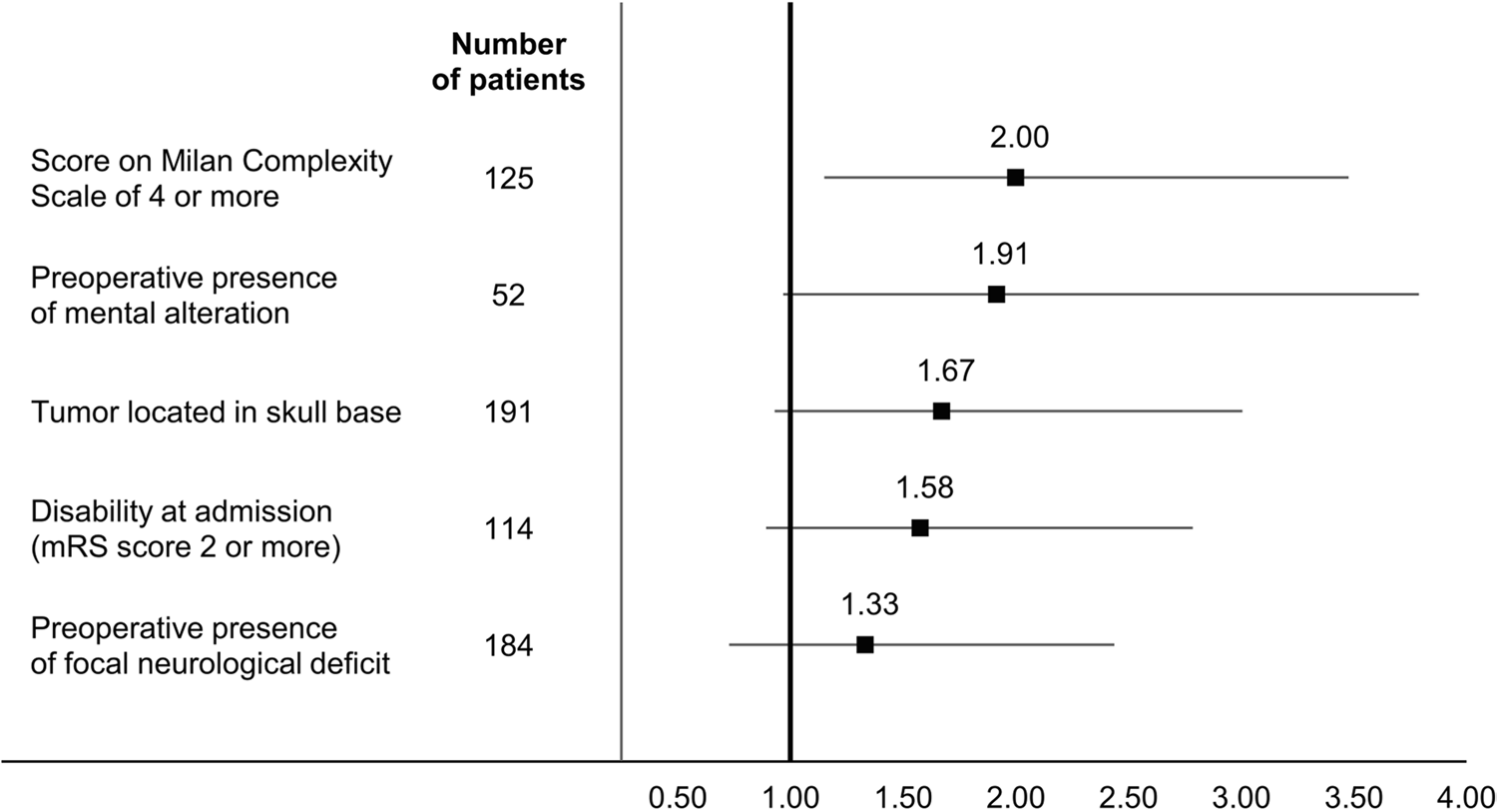
Odds ratios for preoperative risk factors for new focal neurological deficit and other major AEs after meningioma neurosurgery. Abbreviations: mRS, modified Rankin Scale; AEs, adverse events

All the variables included in the multivariate analysis were associated with a higher odds ratio of experiencing a major AE within 3 months of meningioma surgery, but only tumor complexity as assessed by score on the MCS was a statistically significant predictor. An MCS score of 4 or more was associated with a significant increase in OR for major AEs (OR: 2.00, 95% CI: 1.15–3.48) (Fig. 2 “Odds ratios for preoperative risk factors for new focal neurological deficit and other major AEs after meningioma neurosurgery”).

The additional bivariate analysis of the individual variables of the MCS showed a significant association between the two variables “tumor size larger than 4 cm” (*p* < 0.01) and “cranial nerve manipulation” (*p* < 0.001), and the onset of focal neurological deficit or other major AEs. The remaining MCS variables “tumor location in the posterior fossa,” “tumor location in an eloquent area,” and “major brain vessel manipulation” were not significantly associated with the onset of focal neurological deficit or other major AEs in this patient population.

## Discussion

For patients with first-time meningiomas and their physicians, informed decision-making about whether, when, and how to proceed with neurosurgical intervention as the preferred treatment can only be based on data that are available preoperatively. While factors related to the surgical intervention itself, such as surgery duration, extent of resection and tumor histopathology, are relevant for postoperative monitoring, longer-term treatment and follow-up strategies, they are not available until “after the fact.” This study specifically looked at variables available before surgery and identified tumor complexity assessed using the MCS as the most predictive variable of major AE after meningioma surgery.

Our study explicitly only included patients undergoing meningioma surgery for the first time, as these patients represent a distinct population to those undergoing repeat surgery. Unlike other fields in which biopsy is commonly performed before surgery to guide decision-making, the relative inaccessibility of the CNS for simple biopsy means that most first-time meningioma neurosurgeries are performed without knowledge of the exact tumor histology. This contrasts with recurring meningiomas for which histological analysis and tumor grade are generally available from the previous surgery or surgeries. Recurring meningiomas are also significantly more often higher grade [18] and the recurrence itself provides information for treatment decision-making that is not available in the case of first-time meningioma patients.

The definition of major AE used in our study included the onset of new focal neurological deficit. This AE is not adequately captured in treatment-based classification systems such as the system proposed by Ibañez et al. [15] for neurosurgery or the Clavien-Dindo Classification [6] used in general surgery. Given their potential impact on everyday life, new focal neurological deficits may even be of greater concern to patients than other more transient AEs.

Our study included all AEs occurring within 3 months of meningioma surgery. It is well-known that most surgical AEs occur in the immediate postoperative period [30] and this view often guides analyses of AE rates. When looking at AEs of meningioma surgery, including at least one follow-up visit after discharge is likely to help identify AEs of major relevance to the patient for several reasons. Some AEs recorded in the immediate postoperative period may be transient and disappear once tissue reactions related to manipulation have decreased [23]. Other AEs such as wound dehiscence or subdural hematoma may take time to develop or be identified, warranting a longer follow-up period concerning the analysis of surgical AEs.

This study showed a rate of overall major AE of 20.6% and a rate of 12.8% for onset of new focal neurological deficit, which are both in line with recently published studies in the literature [4, 22].

The MCS is based on the study by Ferroli et al. that looked at factors associated with clinical worsening after neurosurgery, including after meningioma surgery and using the Karnofsky Performance Status to capture clinical status [12]. Our study looked at major AE with a definition that included a treatment-based classification system and new focal neurological deficits. Using this definition and a specific population of patients undergoing surgery for meningioma for the first time, our study found the MCS to be the only significant predictor of major AE, confirming the value of the MCS as a preoperative risk assessment.

### Contribution to existing research

Major AEs are common following meningioma surgery [4, 22]. Many studies to-date have identified risk factors [3, 7, 11, 16, 17, 20, 24, 31], but to our knowledge, none have looked specifically at major AE rates in first-time meningioma surgeries, including new focal neurological deficits as major AEs. Our study explicitly analyzed preoperative data available before surgery in order to help the informed guidance of patients before surgery.

This study demonstrated that tumor complexity measured using the MCS is a significant risk factor for major AEs in first-time meningioma surgery. The findings suggest that the MCS should be obtained prior to meningioma surgery to assess the risk of major AE occurrence and should be used in the decision-making process. Interestingly, age was not significantly associated with an increased risk of major AE in our study of first-time meningioma surgeries. Overall, the literature remains inconclusive on this point. The results of some studies suggest elderly patients have a higher risk of AE following meningioma surgery [1, 17], while other studies suggest age alone is not an independent risk factor, but age-associated factors such as comorbidities are [13, 28].

It has to be taken into consideration that the preoperative prediction of the risk for AEs might not only help in the informed guidance of patients and in the decision-making process whether to operate or not, but this prediction might also lead to the identification of patients that should be monitored more closely in the postoperative phase. This could also result in different densities of diagnostic tests in the postoperative phase such as imaging or laboratory tests, tailored to the patient’s preoperative risk stratification. Thereby, such a risk prediction could ultimately help in improving patient care as well as outcome and could also help to focus resources on those patients who might benefit the most.

### Limitations

This study has some limitations that are important to mention. We combined a prospective registry with data that was collected retrospectively. The retrospective data is more likely to be prone to variability and may be subject to confounding. The study design allowed for identification of significant associations but not definite measurement of causation. Furthermore, the definite diagnosis of meningioma requires histopathological analysis, which as mentioned above is not commonly available preoperatively. Ideally, the study would also have included patients with a provisional meningioma diagnosis as their surgery indication, who ultimately received an alternative diagnosis after histopathological analysis of surgery specimen. Identification of these patients was not possible for this study. This effect is estimated as being small however given that provisional diagnosis of meningioma based on imaging is accurate in most cases [33].

### Areas for further research

Further research is warranted to identify and validate preoperative risk factors associated with major AEs in meningioma surgery. Larger studies are required to this end. These studies should ideally include clearly defined patient populations and standardized AE definitions to allow for comparability and meaningful conclusions for neurosurgeons and patients alike.

## Conclusion

High preoperative tumor complexity as measured using the MCS is an independent predictor of the risk of new focal neurological deficits and other major AEs in patients undergoing first-time meningioma surgery.

## Abbreviations

ASA: American Society of Anesthesiologists
CI: Confidence interval
CNS: Central nervous system
ICU: Intensive care unit
IQR: Interquartile range
MCS: Milan Complexity Scale
MRI: Magnetic resonance imaging
mRS: Modified Rankin Scale
OR: Odds ratio
SD: Standard deviation
WHO: World Health Organization
